# Supplementary material for: Phosphoglycerate dehydrogenase activates PKM2 to phosphorylate histone H3T11 and attenuate cellular senescence
Source: Nat Commun. 2023 Mar 10;14:1323. doi: 10.1038/s41467-023-37094-8 (PMC10006232; doi:10.1038/s41467-023-37094-8)
Supplement: Supplementary file 1 — Supplementary Information [file 41467_2023_37094_MOESM1_ESM.pdf]

Supplementary Information

Phosphoglycerate dehydrogenase activates PKM2 to phosphorylate histone H3T11 and attenuate cellular senescence

Yinsheng Wu[1,6], Lixu Tang[2,6], Han Huang[1], Qi Yu[1], Bicheng Hu[3], Gang Wang[1], Feng Ge[4], Tailang Yin[5,*], Shanshan Li[1,*], Xilan Yu[1, *]

[1]State Key Laboratory of Biocatalysis and Enzyme Engineering, School of Life Sciences, Hubei University, Wuhan, Hubei 430062, China

[2]School of Martial Arts, Wuhan Sports University, Wuhan, Hubei 430079, China

[3]The Central Laboratory, Wuhan No.1 Hospital, Wuhan, Hubei 430022, China

[4]Key Laboratory of Algal Biology, Institute of Hydrobiology, Chinese Academy of Sciences, Wuhan, Hubei 430072, China

[5]Reproductive Medicine Center, Renmin Hospital of Wuhan University, Wuhan, Hubei 430060, China

[6]These authors contribute equally to this work

*Corresponding authors: Shanshan Li, shl@hubu.edu.cn

Tailang Yin, reproductive@whu.edu.cn

Xilan Yu, yuxilan@hubu.edu.cn

Supplementary Data:

1. Supplementary (Fig. 1-9).

2. Supplemental Table S1.

Supplementary Fig. 1

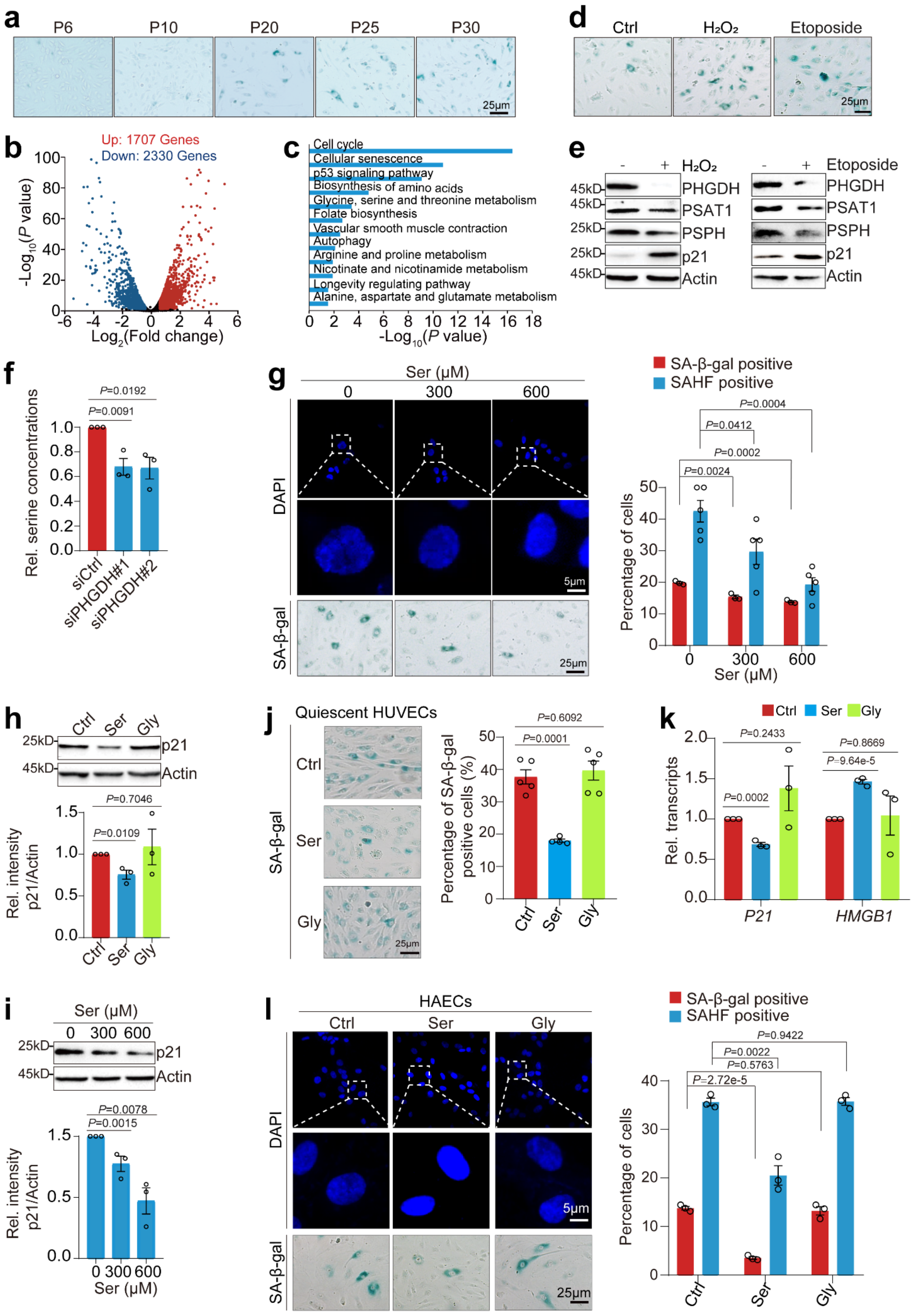

Supplementary Fig. 1 Serine prevents premature cellular senescence.
a SA-β-gal staining of different passages (P6, P10, P20, P25, P30) of HUVECs that continued passaging from the same umbilical cord. **b** Volcano plot of genes differentially expressed between young (P6) and senescent (P20) HUVECs. **c** KEGG analysis of genes differentially expressed in HUVECs during senescence. **d** SA-β-gal staining of H_2O_2-treated and etoposide-treated HUVECs. HUVECs (P10) were treated with 100 μM H_2O_2 for 1 hr. HUVECs (P10) were treated with 50 μM etoposide for 48 hr. **e** Immunoblots analysis the expression of PHGDH, PSAT1 and PSPH in H_2O_2-treated and etoposide-treated HUVECs (P10). p21 was used as a senescence marker. **f** Effect of PHGDH knockdown (siPHGDH#1, siPHGDH#2) on intracellular serine concentrations. HUVECs (P10) were cultured in serine-free medium. **g** Exogenous serine (Ser) attenuates cellular senescence in a dose-dependent manner as determined by SAHF formation (DAPI staining) and SA-β-gal staining. HUVECs (P10) were cultured in serine-free medium and then treated with 300 μM and 600 μM serine for 48 hr. **h** and **i** Exogenous serine (Ser) but not glycine (Gly) reduced the expression of p21. **j** and **k** Exogenous serine (Ser) but not glycine (Gly) attenuates senescence of quiescent HUVECs as determined by SA-β-gal staining (**j**) and transcription of *P21* and *HMGB1* (**k**). HUVECs were cultured in serine-free medium and then treated with 300 μM serine or glycine for 48 hr. **l** Exogenous serine (Ser) but not glycine (Gly) attenuates senescence of HAECs as determined by SAHF formation (DAPI staining) and SA-β-gal staining. HAECs were cultured in serine-free medium and then treated with 300 μM serine or glycine for 48 hr.

For Supplementary Fig. 1**f, h, k-l**, data represent means ± SE; n=3 independent experiments. For Supplementary Fig. 1**g, j**, data represent means ± SE; n=5 independent experiments. Two-sided *t*-tests were used for statistical analysis. For Supplementary Fig. 1**a**, **d**, **e**, data represent a typical example of two biological replicates.

Supplementary Fig. 2

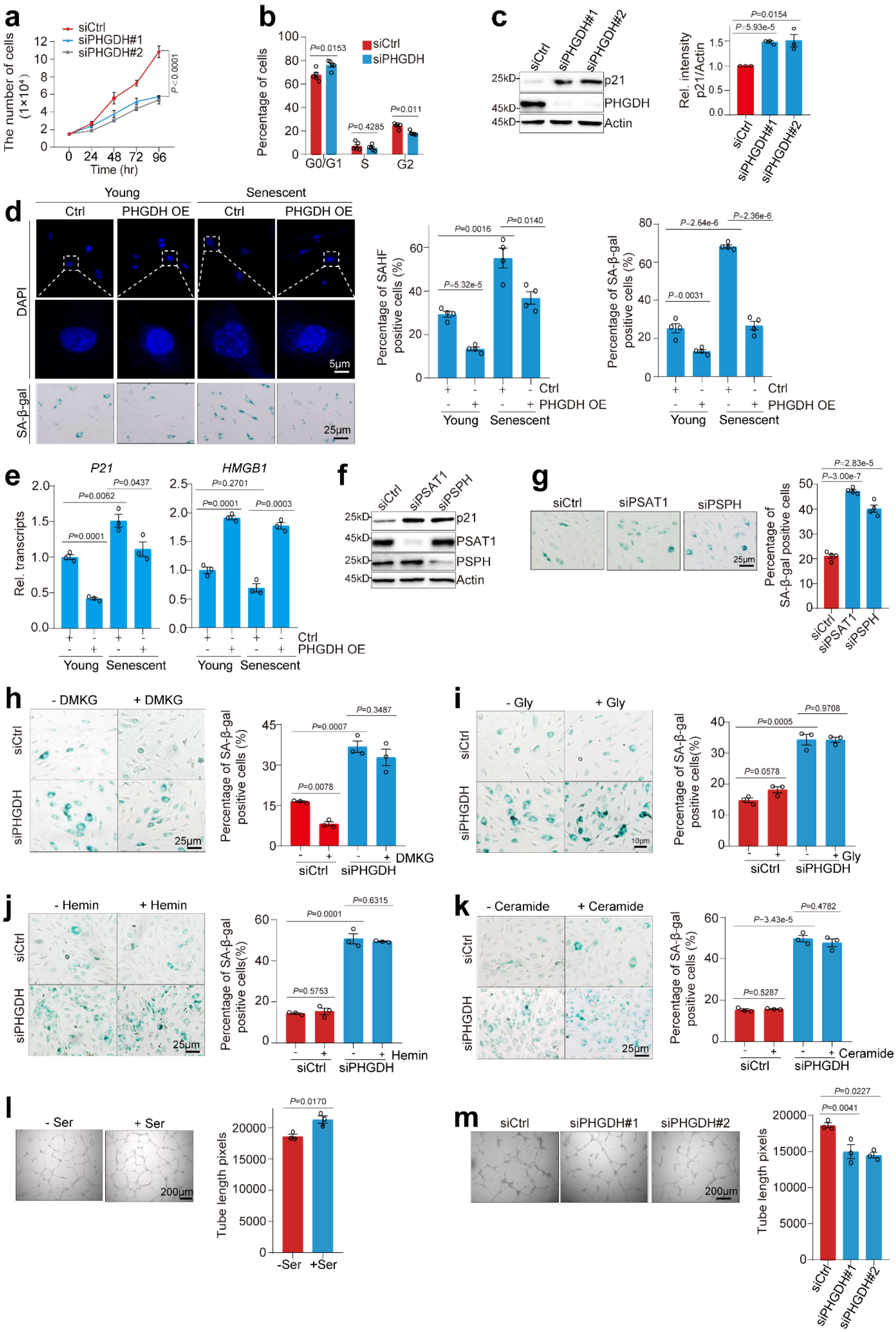

Supplementary Fig. 2 PHGDH-mediated serine biosynthesis prevents premature cellular senescence.

a and **b** Effect of PHGDH knockdown (siPHGDH#1, siPHGDH#2) on the proliferation (**a**) and cell cycle progression (**b**) of HUVECs (P10). **c** Effect of PHGDH knockdown (siPHGDH#1, siPHGDH#2) on p21 expression in HUVECs (P10) as determined by immunoblots. HUVECs (P10) were cultured in serine-free medium. **d** Overexpression of PHGDH (PHGDH OE) alleviated cellular senescence as determined by SAHF formation (DAPI staining) and SA-β-gal staining. HUVECs (young, P10; senescent, P20) were infected with lentivirus to stably express PHGDH. **e** Effect of PHGDH overexpression (PHGDH OE) on *P21* and *HMGB1* transcription in HUVECs (young, P10; senescent, P20) by RT-qPCR analysis. **f-g** Effect of PSAT1 silencing (siPSAT1) and PSPH silencing (siPSPH) on cellular senescence. HUVECs (P10) were cultured in serine-free medium and then transfected with siPSAT1 and siPSPH, respectively. The cellular senescence was indicated by p21 expression (**f**) and SA-β-gal staining (**g**). **h-k** Effect of dimethyl α-ketoglutarate (DMKG) (**h**), glycine (**i**), hemin (**j**), and ceramide (**k**) on senescence of siCtrl and siPHGDH HUVECs as determined by SA-β-gal staining. HUVECs (P10) were grown in serine-free medium and then treated with 300 μM dimethyl α-ketoglutarate (DMKG) for 48 hr, 300 μM glycine (Gly) for 48 hr, 300 μM hemin for 48 hr, 300 μM ceramide for 48 hr. **l** Effect of serine (Ser) on tube formation of HUVECs. HUVECs (P10) were grown in serine-free medium and then treated with 300 μM serine for 48 hr. **m** Effect of PHGDH knockdown (siPHGDH#1, siPHGDH#2) on tube formation of HUVECs (P10) when grown in serine-free medium.

For Supplementary Fig. 2**a**, **c, e, h-m**, data represent means ± SE; n=3 independent experiments. For Supplementary Fig. 2**b**, data represent means ± SE; n=5 independent experiments. For Supplementary Fig. 2**d**, **g**, data represent means ± SE; n=4 independent experiments. Two-sided *t*-tests were used for statistical analysis. For Supplementary Fig. 2**f**, data represent a typical example of two biological replicates.

Supplementary Fig. 3

a

Protein Symbol	Protein Name	Control sample Spectra	Control sample Peptides	Ser sample Spectra	Ser sample Peptides	Enrichment
HSPD1	60 kDa heat shock protein, mitochondrial	16	11	76	36	4.75
EPRS	tRNA ligase	11	12	52	41	4.72
ACLY	ATP-citrate synthase	13	13	58	40	4.46
HSP90B1	Endoplasmin	14	12	44	28	3.14
PKM2	Pyruvate kinase PKM2	42	16	128	45	3.05
IARS	Isoleucine--tRNA ligase, cytoplasmic	11	9	30	26	2.73
IQGAP1	Ras GTPase-activating-like protein	19	16	48	42	2.53
VCP	Transitional endoplasmic reticulum ATPase	23	18	51	33	2.22
GANAB	Neutral alpha-glucosidase AB	21	17	44	29	2.10
P4HB	Protein disulfide-isomerase	25	16	38	23	1.52
HSPA5	78 kDa glucose-regulated protein	47	19	53	26	1.13

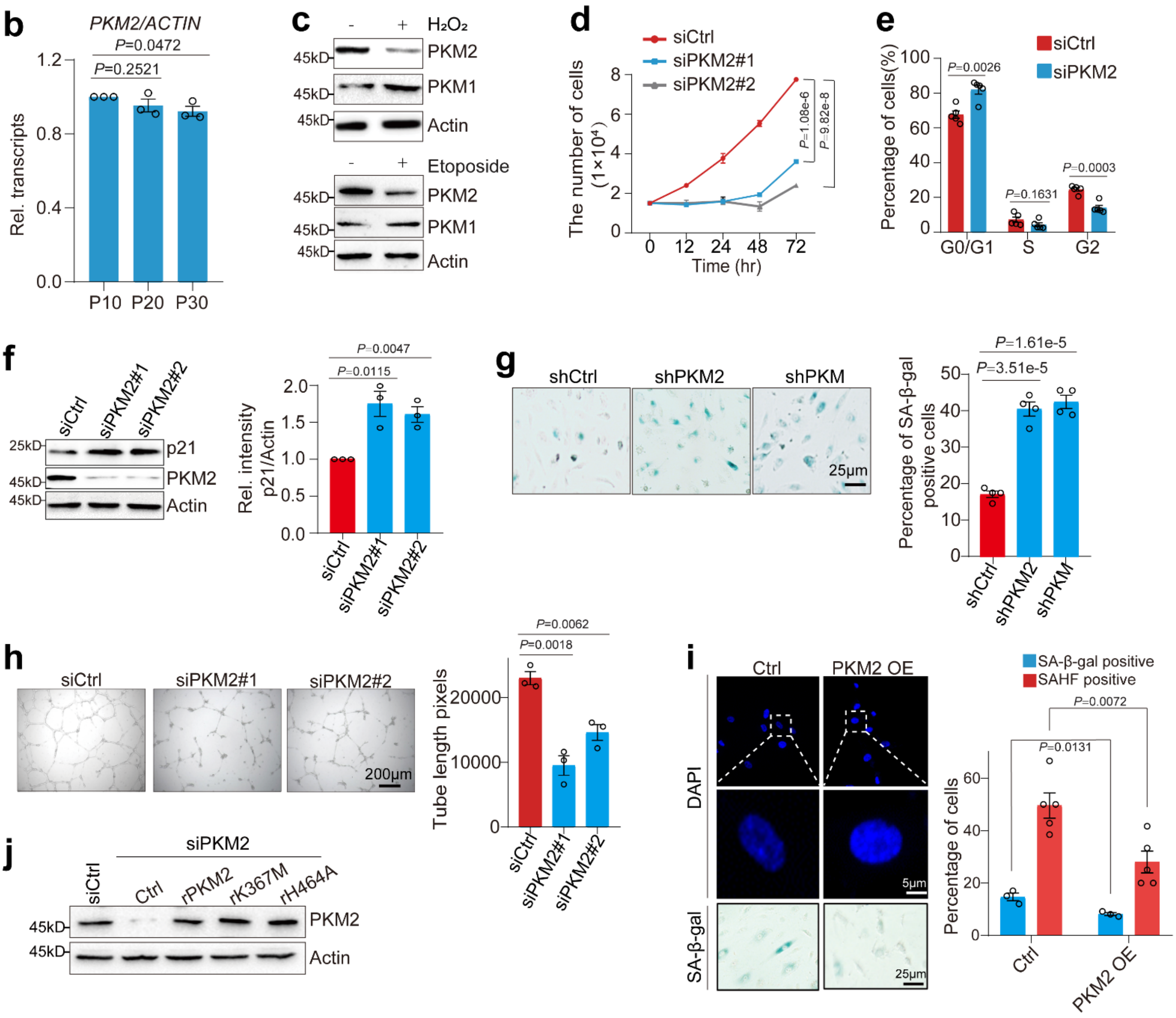

Supplementary Fig. 3 Serine attenuates cellular senescence by targeting PKM2.
a List of potential proteins that are bound by serine as determined by DARTS-MS. The enrichment score was calculated by dividing the spectra of serine-protected samples to the spectra of control samples. In addition, the following criteria was used: the number of peptides in control samples is less than 20; the number of peptides in serine-protected are more than 20. The target candidates were ranked according to their scores. **b** RT-qPCR analysis the transcription of *PKM2* during HUVEC senescence. **c** Immunoblot analysis the expression of PKM1 and PKM2 in H_2O_2-treated and etoposide-treated HUVECs (P10). **d-e** Effect of PKM2 knockdown on HUVECs proliferation (**d**) and cell cycle progression (**e**). HUVECs (P10) were grown in serine-containing medium and then transfected with siPKM2#1 and siPKM2#2. **f** Effect of PKM2 knockdown (siPKM2#1, siPKM2#2) on p21 expression as determined by immunoblots. **g** Effect of PKM2 knockdown (shPKM2) and PKM1/2 knockdown (shPKM) on senescence of HUVECs (P10) as determined by SA-β-gal staining. **h** Effect of PKM2 silencing (siPKM2#1, siPKM2#2) on tube formation of HUVECs (P10). **i** Overexpression of PKM2 (PKM2 OE) alleviated cellular senescence as determined by SAHF formation (DAPI staining) and SA-β-gal staining. HUVECs (P10) were infected with lentivirus to stably PKM2 (PKM2 OE). **j** Effect of overexpression of WT PKM2, PKM2 K367M and PKM2 H464A on p21 expression in HUVECs(P10) in serine-containing medium. HUVECs were infected with lentiviruses to stably overexpression WT PKM2, PKM2 K367M and PKM2 H464A mutants.

For Supplementary Fig. 3**b**, **d**, **f**, **h**, data represent means ± SE; n=3 independent experiments. For Supplementary Fig. 3**e**, **i**, data represent means ± SE; n=5 independent experiments. For Supplementary Fig. 3**g**, data represent means ± SE; n=4 independent experiments. Two-sided *t*-tests were used for statistical analysis. For Supplementary Fig. 3**c**, **j**, data represent a typical example of two biological replicates.

Supplementary Fig. 4

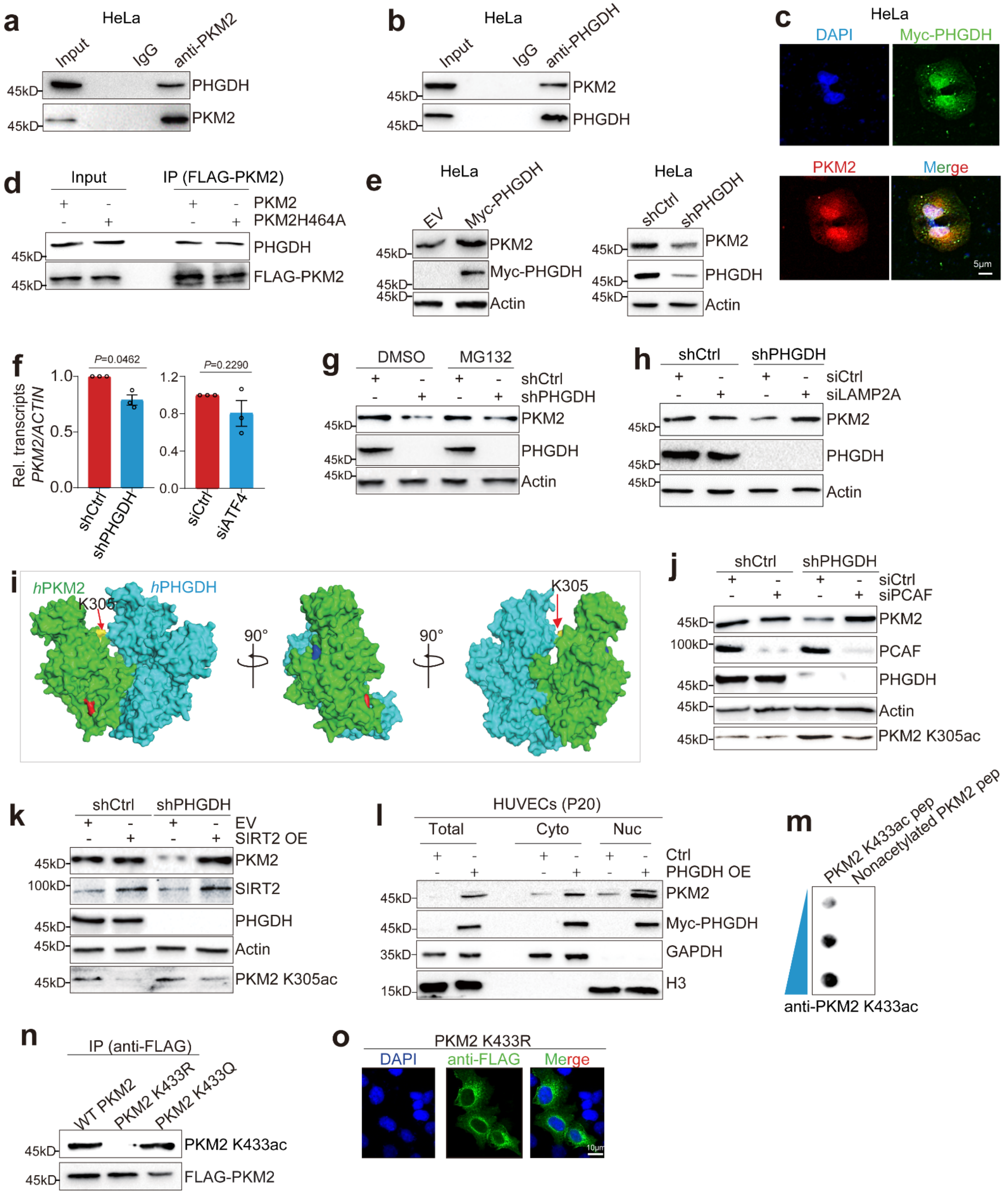

Supplementary Fig. 4 PHGDH directly binds and stabilizes PKM2.

a and **b** PHGDH interacts with PKM2 as determined by Co-IP (**a**) and reciprocal IP (**b**). PKM2 and PHGDH were immunoprecipitated from HeLa cells by anti-PKM2 and anti-PHGDH antibodies, respectively. **c** Immunofluorescence assay showing PHGDH co-localized with PKM2. As the anti-PHGDH antibody and anti-PKM2 antibody were rabbit antibodies, HeLa cells were transfected with the plasmid expressing Myc-PHGDH and Myc-PHGDH was detected with anti-Myc antibody. PKM2 was detected with anti-PKM2 antibody. Blue: DAPI; Green: PHGDH; Red: PKM2. **d** Co-IP assay showing mutation of PKM2 H464A did not affect its interaction with PHGDH. **e** Immunoblot analysis the expression of PKM2 in PHGDH overexpression (PHGDH OE) and PHGDH knockdown (shPHGDH) HeLa cells. EV, empty vector. **f** RT-qPCR analysis the transcription of *PKM2* in control (shCtrl, siCtrl), PHGDH-knockdown (shPHGDH) and ATF4-knockdown (siATF4) HUVECs. **g** Immunoblot analysis the expression of PKM2 in control (shCtrl) and PHGDH-knockdown (shPHGDH) HUVECs when treated with DMSO or 10 μM MG132. **h** Immunoblot analysis the expression of PKM2 in control (shCtrl) and PHGDH-knockdown (shPHGDH) HUVECs when transfected with siCtrl or siLAMP2A. **i** Molecular docking of PHGDH and PKM2. The PKM2 K305 acetylation site was highlighted in yellow. **j** Knockdown of PCAF (siPCAF) rescued the reduced PKM2 in shPHGDH HUVECs. **k** Overexpression of SIRT2 (Myc-SIRT2) rescued the reduced PKM2 in shPHGDH cells. The shPHGDH HUVECs were transfected with pCMV or pCMV-SIRT2. **l** Overexpression of PHGDH in HUVECs (P20) promoted the nuclear translocation of PKM2. **m** Dot blot analysis showing the specificity of anti-PKM2 K433ac antibody using different diluted PKM2 K433ac peptide (PKM2 K433ac pep) and PKM2 K433 peptide (nonacetylated PKM2 pep). **n** Validation the specificity of anti-PKM2 K433ac antibody. HeLa cells were transfected with plasmids that express FLAG-WT PKM2, FLAG-PKM2 K433R, and FLAG-PKM2 K433Q, which were then immunoprecipitated with anti-FLAG antibody. **o** Immunofluorescence analysis the subcellular localization of PKM2 K433R in HUVECs. HUVECs were infected with lentivirus to overexpress FLAG-PKM2 K433R. PKM2 K433R was detected with anti-FLAG antibody.

For Supplementary Fig. 4**f**, data represent means ± SE; n=3 independent experiments. Two-sided *t*-tests were used for statistical analysis. For Supplementary Fig. 4**a-e**, **g**, **h**, **j-o**, data represent a typical example of two biological replicates.

Supplementary Fig. 5

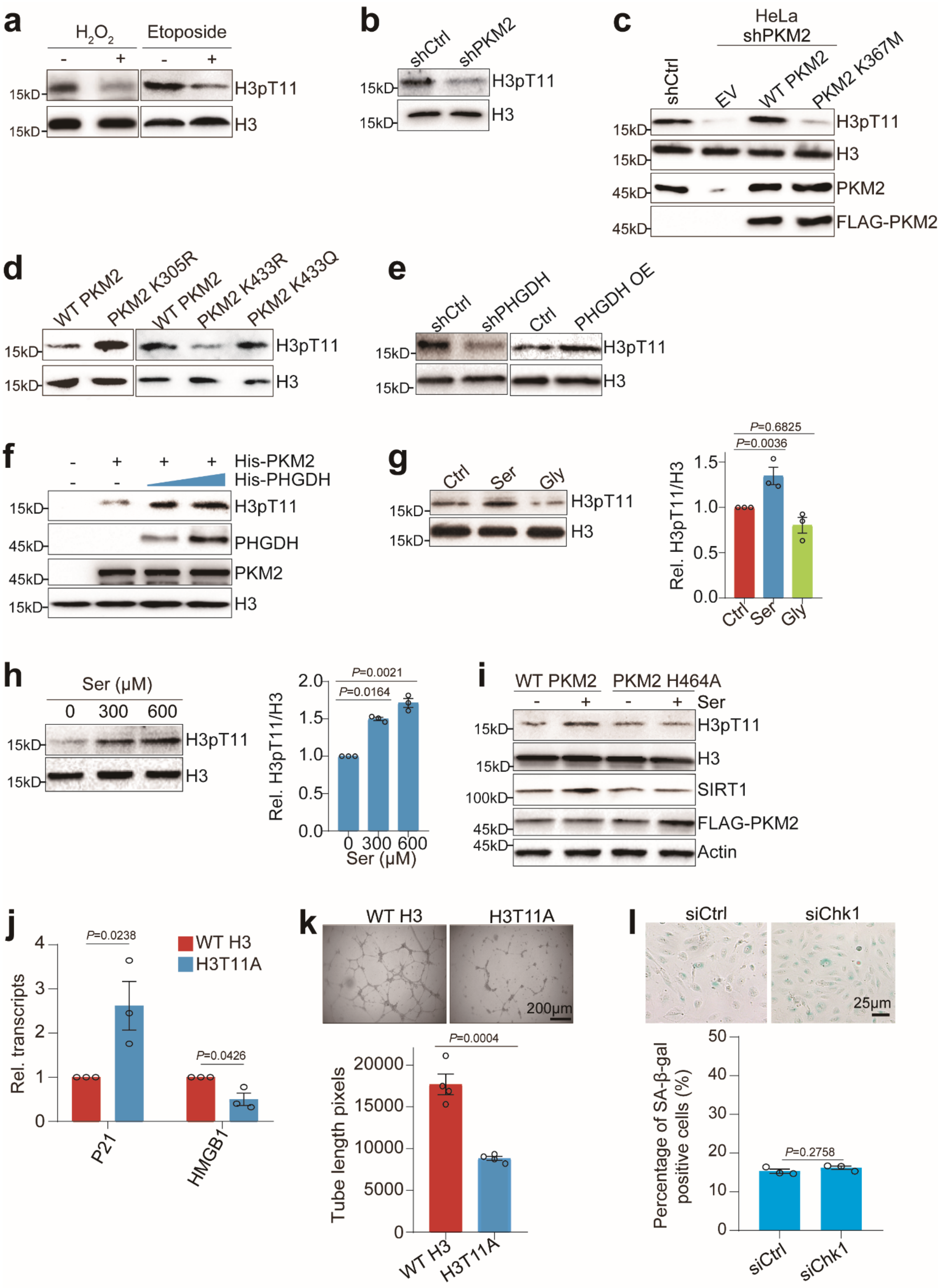

Supplementary Fig. 5 PHGDH stimulates the activity of PKM2 to phosphorylate histone H3T11. **a** Immunoblot analysis of H3pT11 in H_2O_2-treated and etoposide-treated HUVECs (P10). **b** Immunoblot analysis of H3pT11 in shCtrl and shPKM2 HUVECs. **c** Overexpression of WT PKM2 but not PKM2 K367M mutant rescued the reduced H3pT11 in PKM2-knockdown (shPKM2) HeLa cells. Control (shCtrl) and shPKM2 HeLa cells when transfected with plasmids that overexpress shPKM2-resistant WT PKM2 and PKM2 K367M. EV, empty vector. **d** Immunoblot analysis of H3pT11 in HUVECs (P10) infected with lentiviruses that overexpress WT PKM2, PKM2K305R, PKM2K433R, and PKM2K433R. **e** Immunoblot analysis of H3pT11 in HUVECs (P10) infected with lentiviruses to stably knock down PHGDH (shPHGDH) and overexpress PHGDH (PHGDH OE). **f** *In vitro* kinase assay showed that the purified recombinant PHGDH (His-PHGDH) directly stimulates the activity of recombinant PKM2 (His-PKM2) to phosphorylate H3T11. **g** Exogenous serine (Ser) but not glycine (Gly) significantly increased the intracellular H3pT11. HUVECs (P10) were cultured in serine-free medium and then treated with 300 μM serine or glycine for 48 hr. **h** Exogenous serine (Ser) induced the intracellular H3pT11 in a dose-dependent manner. HUVECs (P10) were cultured in serine-free medium and then treated with 0-600 μM serine for 48 hr. **i** Serine (Ser) induced H3pT11 in HUVECs that were infected with lentiviruses that overexpress WT PKM2 but not PKM2 H464A. HUVECs were cultured in serine-free medium and then treated with 300 μM serine. **j** qRT-PCR analysis the transcription of *P21* and *HMGB1* in HUVECs infected with lentiviruses that overexpress WT H3 and H3T11A mutant. **k** Effect of overexpression of WT H3 and H3T11A on tube formation of HUVECs. **l** Effect of Chk1 knockdown (siChk1) on cellular senescence as indicated by SA-β-gal staining. HUVECs (P10) were grown in serine-containing medium and then transfected with siChk1.

For Supplementary Fig. 5**g**, **h**, **j**, **l**, data represent means ± SE; n=3 independent experiments. For Supplementary Fig. 5**k**, data represent means ± SE; n=4 independent experiments. Two-sided *t*-tests were used for statistical analysis. For Supplementary Fig. 5**a**-**f**, **i**, data represent a typical example of two biological replicates.

Supplementary Fig. 6

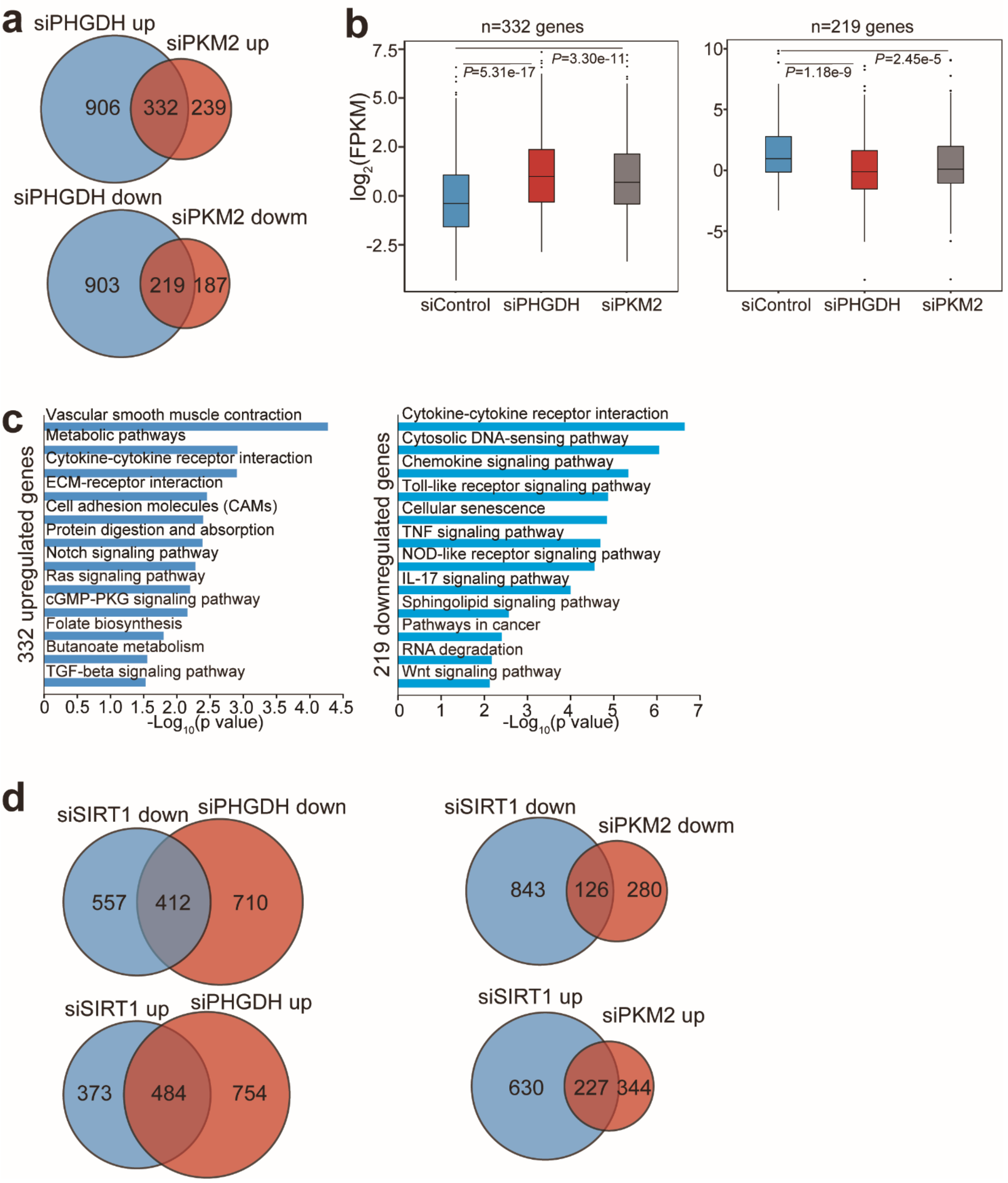

Supplementary Fig. 6 PHGDH and PKM2 co-regulate the expression of senescence-associated genes.

a Venn diagrams showing the overlap between genes regulated by PHGDH and PKM2. **b** Box plots showing 332 genes were co-upregulated and 219 genes were co-downregulated by PHGDH and PKM2, respectively. Centre lines denote medians; box limits denote 25th and 75th percentiles; whiskers denote maxima and minima. Two-sided Wilcoxon test in R (package ggpval) was used for statistical analysis. FPKM, fragments per kilobase of exon per million fragments mapped. Three biological independent replicates were performed. **c** Left panel: KEGG analysis of 332 genes co-upregulated by PKM2 and PHGDH. Right panel: KEGG analysis of 219 genes co-downregulated by PKM2 and PHGDH. **d** Left panel: Venn diagrams showing the overlap between genes regulated by SIRT1/PHGDH. Right panel: Venn diagrams showing the overlap between genes regulated by SIRT1/PKM2.

Supplementary Fig. 7

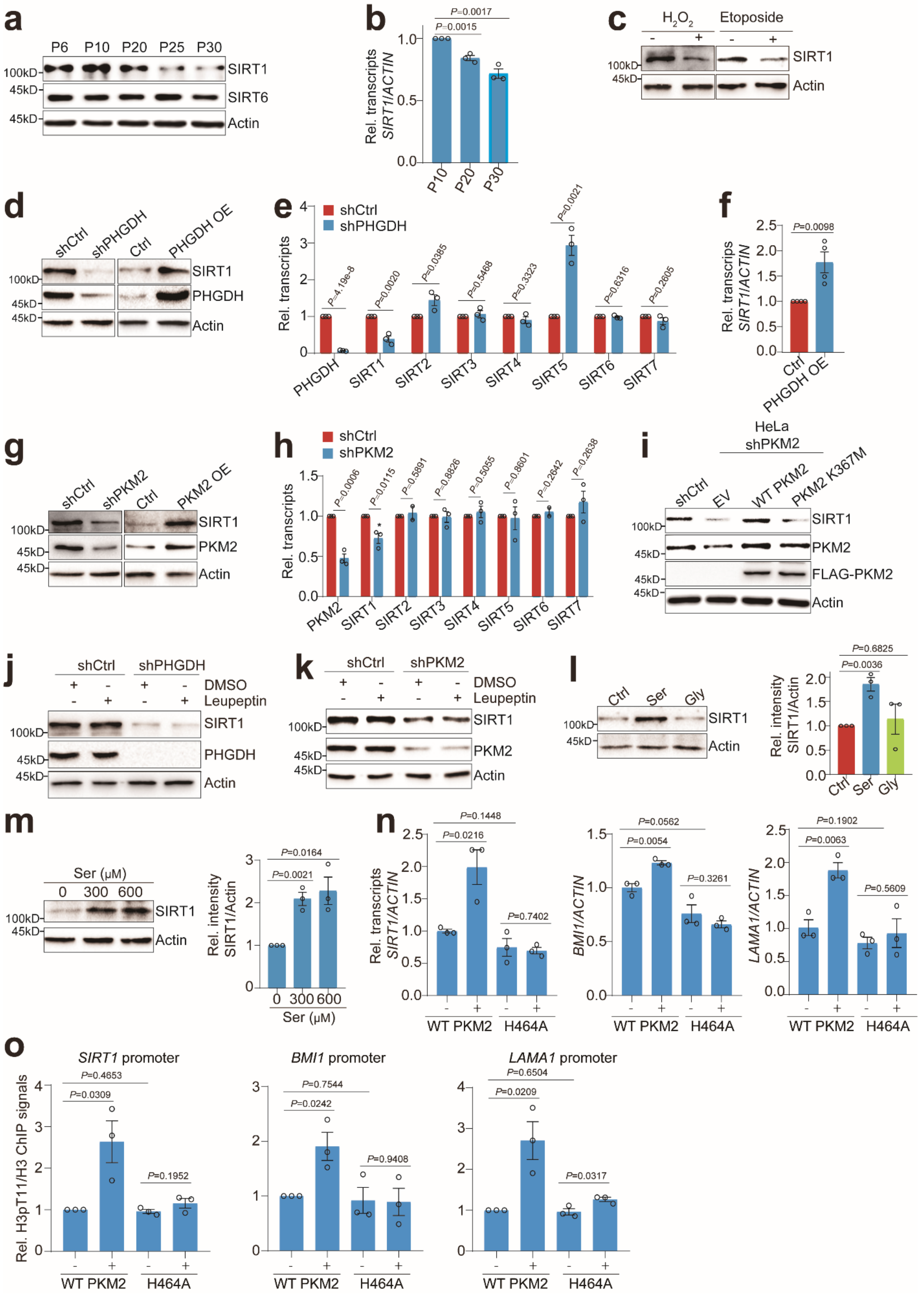

Supplementary Fig. 7 PHGDH and PKM2 promote the transcription of senescence-associated genes.

a Immunoblot analysis the expression of SIRT1 and SIRT6 in HUVECs during cellular senescence. **b** RT-qPCR analysis the transcription of *SIRT1* during HUVEC senescence. **c** Immunoblot analysis the expression of SIRT1 in H_2O_2-treated and etoposide-treated HUVECs (P10). **d** Effect of PHGDH knockdown (shPHGDH) and overexpression (PHGDH OE) on SIRT1 expression in HUVECs (P10) when cultured in serine-free medium. **e** Effect of PHGDH knockdown (shPHGDH) on the transcription of *SIRT1*-*SIRT7* in HUVECs(P10) when cultured in serine-free medium. **f** Lentiviral-mediated overexpression of PHGDH significantly increased SIRT1 transcription in HUVECs (P10) when cultured in serine-free medium. **g** Effect of PKM2 knockdown (shPKM2) and overexpression (PKM2 OE) on SIRT1 expression in HUVECs (P10). **h** Effect of PKM2 knockdown (shPKM2) on the transcription of *SIRT1*-*SIRT7* in HUVECs. **i** Immunoblot analysis the expression of SIRT1 in control (shCtrl) and shPKM2 HeLa cells when transfected with plasmids that overexpress shPKM2-resistant WT PKM2 and PKM2 K367M. **j** The reduced SIRT1 in PHGDH knockdown (shPHGDH) HUVECs cannot be rescued by leupeptin treatment. HUVECs (P10) were cultured in serine-free medium and then treated with 20 μM leupeptin. **k** The reduced SIRT1 in PKM2 knockdown (shPKM2) HUVECs cannot be rescued by leupeptin treatment. **l** Exogenous serine (Ser) but not glycine (Gly) increased the expression of SIRT1. HUVECs (P10) were cultured in serine-free medium and then treated with 300 μM serine (Ser) or glycine (Gly) for 48 hr. **m** Serine (Ser) induced the expression of SIRT1 in a dose-dependent manner in HUVECs. **n** RT-qPCR analysis the effect of serine (Ser) on transcription of *SIRT1*, *BMI1* and *LAMA1* in HUVECs that stably express WT PKM2 and PKM2 H464A. **o** ChIP-qPCR analysis of H3pT11 on the promoters of *SIRT1*, *BMI1* and *LAMA1* in HUVECs that stably express WT PKM2 and PKM2 H464A.

For Supplementary Fig. 7**b**, **e**, **h**, **l**, **m**-**o**, data represent means ± SE; n=3 independent experiments. For Supplementary Fig. 7**f**, data represent means ± SE; n=4 independent experiments. Two-sided *t*-tests were used for statistical analysis. For Supplementary Fig. 7**a**, **c**, **d**, **g**, **i**-**k**, data represent a typical example of two biological replicates.

Supplementary Fig. 8

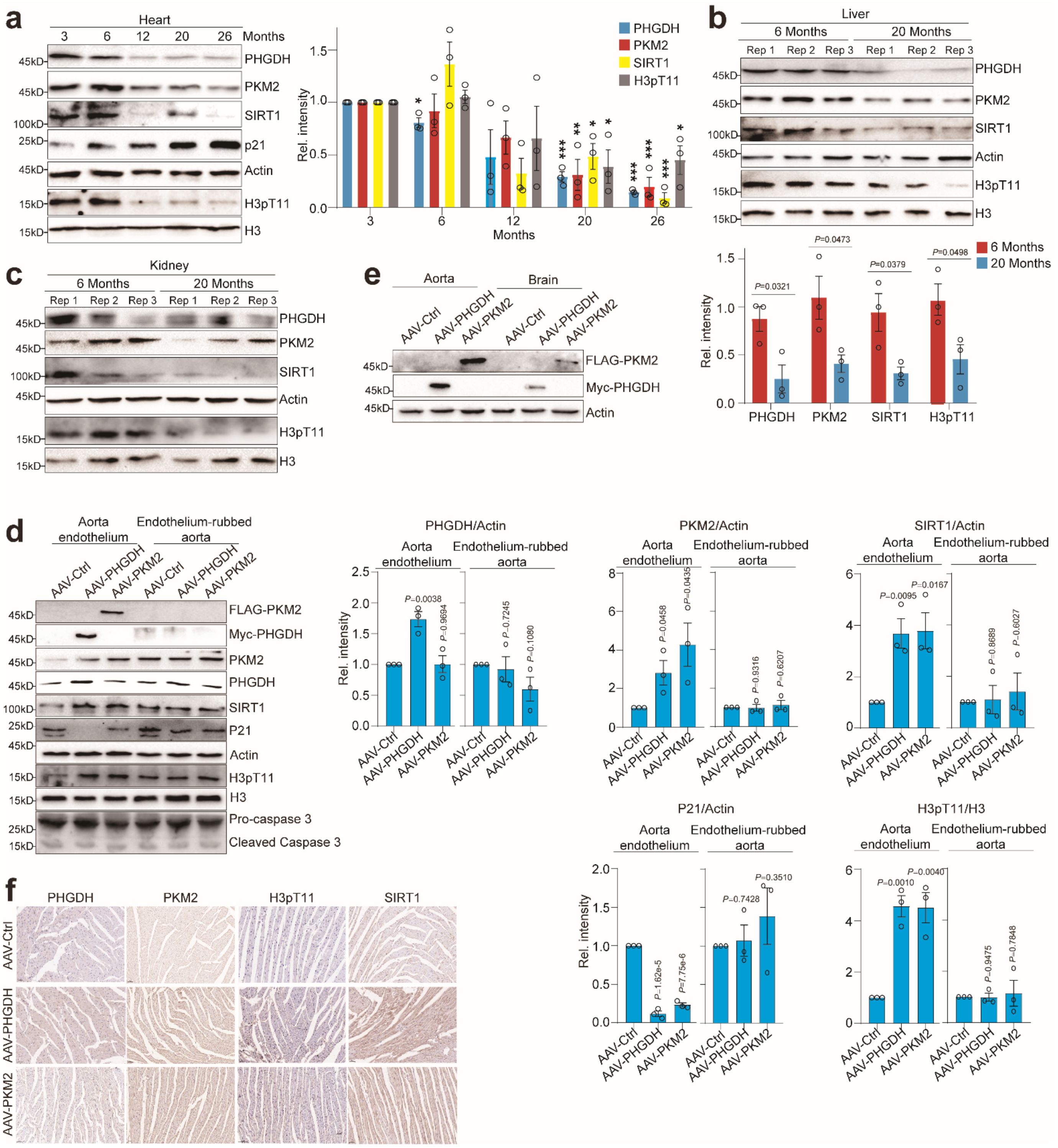

Supplementary Fig. 8 PHGDH and PKM2 regulate ageing phenotypes in mice.
a Immunoblots of PHGDH, PKM2, SIRT1, p21 and H3pT11 levels in the heart tissue of naturally aged mice (3-26 months). **b-c** Immunoblots of PHGDH, PKM2, SIRT1, p21 and H3pT11 levels in the liver (**b**) and kidney (**c**) of young mice (6 months) and old mice (20 months). **d** The aortae were taken from AAV-Ctrl, AAV-PHGDH and AAV-PKM2 mice. The endothelium was rubbed from the aortae. The aorta endothelium and endothelium-rubbed aortae were then subjected to immunoblots of FLAG-PKM2, FLAG-PHGDH, PHGDH, PKM2, SIRT1, p21, H3pT11, Pro-caspase 3 and Cleaved caspase 3. **e** Immunoblots of FLAG-PKM2 and FLAG-PHGDH in the aorta and brain samples of AAV-Ctrl, AAV-PHGDH and AAV-PKM2 mice. **f** Representative immunohistochemical staining of PHGDH, PKM2, H3pT11 and SIRT1 in the heart samples of AAV-Ctrl, AAV-PHGDH and AAV-PKM2 mice.

For Supplementary Fig. 8**a**, **b**, data represent means ± SE; n=3 independent experiments. Two-sided *t*-tests were used for statistical analysis. For Supplementary Fig. 8**e**, **f**, data represent a typical example of three biological replicates.

Supplementary Fig. 9

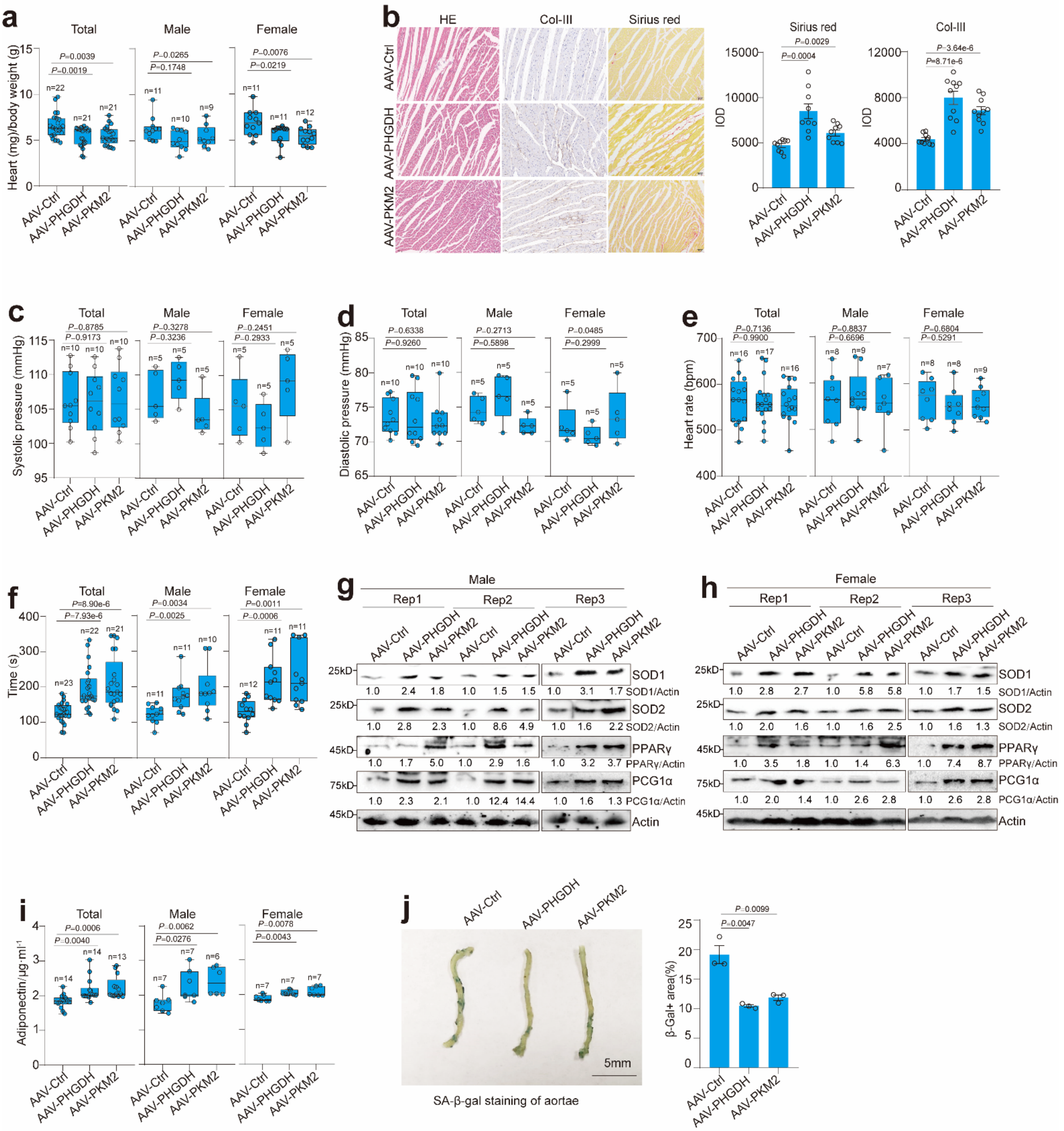

Supplementary Fig. 9 PHGDH and PKM2 regulate muscle activity in mice.
a Analysis of heart/body weight in AAV-Ctrl, AAV-PHGDH and AAV-PKM2 mice. **b** Representative immunohistochemical staining of the cardiac structure and collagen content of AAV-Ctrl, AAV-PHGDH and AAV-PKM2 mice. Col-III antibody and Sirius red were used to stain collagen in the hearts. **c-d** Analysis of blood pressure in AAV-Ctrl, AAV-PHGDH and AAV-PKM2 mice. **e** Analysis of heart rate in AAV-Ctrl, AAV-PHGDH and AAV-PKM2 mice. **f** Total time achieved by AAV-Ctrl, AAV-PHGDH and AAV-PKM2 mice in a treadmill running performance test. **g-h** Immunoblots of SOD1, SOD2, PPARγ and PGC-1α in the muscle of AAV-Ctrl, AAV-PHGDH and AAV-PKM2 male (**g**) and female (**h**) mice. **i** Analysis of plasma adiponectin in AAV-Ctrl, AAV-PHGDH and AAV-PKM2 mice. **j** SA-β-gal staining of the aortae from AAV-Ctrl, AAV-PHGDH and AAV-PKM2 mice.

For Supplementary Fig. 9**a**, **c-f**, **i**, data represent means ± SE; the number of total mice, male mice and female mice was indicated in the graphs, centre lines denote medians, box limits denote 25th and 75th percentiles and whiskers denote maximum and minimum values. Two-sided *t*-tests were used for statistical analysis. For Supplementary Fig. 9**b**, data represent means ± SE, n=9. Two-sided *t*-tests were used for statistical analysis. For Supplementary Fig. 9**j**, data represent means ± SE; n=3 independent experiments. Two-sided *t*-tests were used for statistical analysis.

Supplementary Table 1 List of oligonucleotides used in this study

Gene name	Sequence
**ChIP-qPCR**	
SIRT1 promoter	AGAAACGCTGTGCTCCAGGCAGATG
	GTAAAACGAGGGGTACCTAGTAGTTC
LAMA1 promoter	TGGTGGGATGATAATGAGATATTGTCC
	CTGAGGCAGGAGAATTGCTTGAAC
BMI1 promoter	GGCCTGACTACACCGACACT
	CTCCAAAATGGCTCGGAGT
**qRT-PCR**	
ACTIN	GCCGACAGGATGCAGAAGGAGATCA
	AAGCATTTGCGGTGGACGATGGA
PHGDH	ATCTCTCACGGGGGTTGTG
	AGGCTCGCATCAGTGTCC
PSAT1	GTCCAGTGGAGCCCCAAAA
	TGCCTCCCACAGACCTATGC
PSPH	CACGGTCATCAGAGAAGAAG
	GGTTGCTCTGCTATGAGTCT
PKM2	GCTGCCATCTACCACTTGC
	CCAGACTTGGTGAGGACGATT
SIRT1	TGCTGGCCTAATAGAGTGGC
	CCTCAGCGCCATGGAAAATG
HIST1H3H	AAGCGGGTGACTATCATGC
	GGGTATGTAATCAGTGAGTCCTTG
BMI1	CTTTCATTGTCTTTTCCGCCC
	AAGTACCCTCCACAAAGCAC
LAMA1	AATGGAGTGAGACAGGAACAAG
	TGACCATACGATGCCTTGATG
mACTIN	GTGACGTTGACATCCGTAAAGA
	GCCGGACTCATCGTACTCC
mPHGDH	GAAATCGCAGTCCAGTTTGTG
	TGCCAGACCAATCCAAGG
mPKM2	GTCTGGAGAAACAGCCAAGG
	CGGAGTTCCTCGAATAGCTG
mSIRT1	CTCTGAAAGTGAGACCAGTAGC
	TGTAGATGAGGCAAAGGTTCC
ATF4	TTCTCCAGCGACAAGGCTAAGG
	CTCCAACATCCAATCTGTCCCG
P21	GACACCACTGGAGGGTGACT
	CAGGTCCACATGGTCTTCCT
HMGB1	GCGAAGAAACTGGGAGAGATGTG
	GCATCAGGCTTTCCTTTAGCTCG
mATF4	ATGGCGTATTAGAGGCAGC

	CTTTGTCCGTTACAGCAACAC
