# Supplementary material for: Phosphoglycerate dehydrogenase activates PKM2 to phosphorylate histone H3T11 and attenuate cellular senescence
Source: Nat Commun. 2023 Mar 10;14:1323. doi: 10.1038/s41467-023-37094-8 (PMC10006232; doi:10.1038/s41467-023-37094-8)
Supplement: Supplementary file 3 — Reporting Summary [file 41467_2023_37094_MOESM3_ESM.pdf]

nature portfolio

Corresponding author(s): Shanshan Li, Xilan Yu, Tailang Yin

Last updated by author(s): 2/7/2013

Reporting Summary

Nature Portfolio wishes to improve the reproducibility of the work that we publish. This form provides structure for consistency and transparency in reporting. For further information on Nature Portfolio policies, see our Editorial Policies and the Editorial Policy Checklist.

Statistics

For all statistical analyses, confirm that the following items are present in the figure legend, table legend, main text, or Methods section.

n/a	Confirmed	
☐	☒	The exact sample size (*n*) for each experimental group/condition, given as a discrete number and unit of measurement
☐	☒	A statement on whether measurements were taken from distinct samples or whether the same sample was measured repeatedly
☐	☒	The statistical test(s) used AND whether they are one- or two-sided<br>*Only common tests should be described solely by name; describe more complex techniques in the Methods section.*
☒	☐	A description of all covariates tested
☒	☐	A description of any assumptions or corrections, such as tests of normality and adjustment for multiple comparisons
☐	☒	A full description of the statistical parameters including central tendency (e.g. means) or other basic estimates (e.g. regression coefficient) AND variation (e.g. standard deviation) or associated estimates of uncertainty (e.g. confidence intervals)
☐	☒	For null hypothesis testing, the test statistic (e.g. *F*, *t*, *r*) with confidence intervals, effect sizes, degrees of freedom and *P* value noted<br>*Give P values as exact values whenever suitable.*
☒	☐	For Bayesian analysis, information on the choice of priors and Markov chain Monte Carlo settings
☐	☒	For hierarchical and complex designs, identification of the appropriate level for tests and full reporting of outcomes
☒	☐	Estimates of effect sizes (e.g. Cohen's *d*, Pearson's *r*), indicating how they were calculated

Our web collection on statistics for biologists contains articles on many of the points above.

Software and code

Policy information about availability of computer code

Data collection	LabChart(v.8)(https://www.adinstruments.com.cn/products/labchart)
Data analysis	No custom software or algorithm was used in this study. All software used in this study for data analysis are either commercially available or open source.<br>Image J (v1.8.0) (https://imagej.en.softonic.com/)<br>prism(v.8.0.1) (https://www.graphpad.com/scientific-software/prism/)<br>SRA toolkit (v.2.9.2)(https://www.ncbi.nlm.nih.gov/sra/docs/toolkitsoft/)<br>Fastqc (v.0.11.4 & v.0.11.9) (http://www.bioinformatics.babraham.ac.uk/projects/fastqc/)<br>Trim Galore (v.0.3.1 & v.2.11) (https://github.com/FelixKrueger/TrimGalore)<br>Bowtie2 (v.2.1.0) (https://www.uio.no/english/services/it/research/hpc/abel/help/software/bowtie2.html)<br>Samtools (1.7-2) (http://samtools.sourceforge.net/)<br>MACS2 (v.2.1.1) (https://taoliu.github.io/MACS/)<br>Bedtools (v.2.19.0) (https://sourceforge.net/projects/bedtools/)<br>R (v.3.1) (https://cran.rstudio.com/)<br>IGV (v.2.0) (http://www.igv.org/)<br>SPSS (https://www.ibm.com/analytics/academic-statistical-software)<br>KOBAS 3.0 (http://kobas.cbi.pku.edu.cn/)<br>cutadapt (v.1.16) (http://cutadapt.readthedocs.io/)<br>hisat2 (v.2.1.0) (https://ccb.jhu.edu/software/hisat2/index.shtml)<br>edgeR (v.3.24) (http://www.bioconductor.org/packages/release/bioc/html/edgeR.htm)<br>DEGSeq package (v.21.24.0) (http://bioconductor.org/packages/3.9/bioc/html/DESeq2.html)

deepTools (v.2.0)(https://github.com/deeptools/deepTools/archive/1.5.12.tar.gz)
SWISS-MODEL (https://swissmodel.expasy.org)
AutoDock Vina(1.5.6) (https://vina.scripps.edu/)
PyMOL(2.2.0)(http://pymol.prg/2/)
Image Pro Plus 6.0(https://www.meyerinst.com/mediacybernetics/image-pro-plus/)

For manuscripts utilizing custom algorithms or software that are central to the research but not yet described in published literature, software must be made available to editors and reviewers. We strongly encourage code deposition in a community repository (e.g. GitHub). See the Nature Portfolio guidelines for submitting code & software for further information.

Data

Policy information about availability of data

All manuscripts must include a data availability statement. This statement should provide the following information, where applicable:

- Accession codes, unique identifiers, or web links for publicly available datasets
- A description of any restrictions on data availability
- For clinical datasets or third party data, please ensure that the statement adheres to our policy

Data availability.
All RNA-seq data in this study were deposited on GEO database. The GEO accession number for the raw RNA-seq data (young HUVECs and senescent HUVECs) in this manuscript is PRJNA764604 [https://www.ncbi.nlm.nih.gov/bioproject/PRJNA764604]. The GEO accession number for the raw RNA-seq data set (PKM2 knockdown, PHGDH knockdown, SIRT1 knockdown) in this manuscript is PRJNA673282 [https://www.ncbi.nlm.nih.gov/bioproject/PRJNA673282] and PRJNA766438 [https://www.ncbi.nlm.nih.gov/bioproject/PRJNA766438]. The mass spectrometry proteomics data have been deposited to the ProteomeXchange Consortium via the PRIDE partner repository with the dataset identifier PXD035586 and PXD035585. The structure of PKM2 and p300 was obtained from the PDB (PDB: 1ZJH, 4PZT). Source data are provided with this paper.

Human research participants

Policy information about studies involving human research participants and Sex and Gender in Research.

Reporting on sex and gender	This study did not involve human research. n/a
Population characteristics	n/a
Recruitment	n/a
Ethics oversight	n/a

Note that full information on the approval of the study protocol must also be provided in the manuscript.

Field-specific reporting

Please select the one below that is the best fit for your research. If you are not sure, read the appropriate sections before making your selection.

☒ Life sciences ☐ Behavioural & social sciences ☐ Ecological, evolutionary & environmental sciences

For a reference copy of the document with all sections, see nature.com/documents/nr-reporting-summary-flat.pdf

Life sciences study design

All studies must disclose on these points even when the disclosure is negative.

Sample size	Sample sizes were determined based on previous experience or similar published studies. To determine whether the outcome is statistically significant, at least three biological independent replicates were performed for each experiment. For other experiments, to determine the outcome is reproducible, at least 2-3 biological replicates were performed for each experiment. Related reference: Molecular Cell, 2015, 60:408-421; Nature Metabolism, 2021, 3(7):983-1000.
Data exclusions	No data were excluded from this study.
Replication	We confirmed that all attempts to replicate experiments were successful. For quantification of serine and glycine, qRT-PCR, ChIP-qPCR, tube formation assay, SA-β-gal staining and SAHF experiments data are derived from 3 biological replicates. For Co-IP and immunoblots experiments were performed with at least two biological replicates. For grip strength test, treadmill performance test, and echocardiographic imaging, experiments were performed with at least 6 biological replicates.
Randomization	The allocation of samples and animals into different groups was random.
Blinding	For animal experiments, the researchers performed the experiments in a double-blinded manner. For other experiments, it was necessary for

Blinding

the researchers to be aware of the treatment applied and mutants used. Meanwhile, the researchers also need to use appropriate controls in each experiment.

Reporting for specific materials, systems and methods

We require information from authors about some types of materials, experimental systems and methods used in many studies. Here, indicate whether each material, system or method listed is relevant to your study. If you are not sure if a list item applies to your research, read the appropriate section before selecting a response.

Materials & experimental systems

n/a	Involved in the study	
☐	☒	Antibodies
☐	☒	Eukaryotic cell lines
☒	☐	Palaeontology and archaeology
☐	☒	Animals and other organisms
☒	☐	Clinical data
☒	☐	Dual use research of concern

Methods

n/a	Involved in the study	
☒	☐	ChIP-seq
☒	☐	Flow cytometry
☒	☐	MRI-based neuroimaging

Antibodies

Antibodies used

Commercially available antibodies:
Rabbit anti-histone H3 (ab1791; Abcam)
Rabbit anti-histone H3pT11 (ab5168; Abcam)
Mouse anti-FLAG M2 (F1804-1MG; Sigma-aldrich)
Mouse anti-Myc (60003-2-Ig; proteintech)
Rabbit anti-PHGDH (14719-1-AP; Proteintech)
Rabbit anti-GAPDH (10494-1-AP; proteintech)
Rabbit anti-PSAT1 (10501-1-AP; proteintech)
Rabbit anti-PSPH (14513-1-AP; proteintech)
Rabbit anti-PKM1 (15821-1-AP; proteintech)
Rabbit anti-p21 (10355-1-AP; proteintech)
Rabbit anti-PCAF (13983-1-AP; proteintech)
Rabbit anti-SIRT1 (13161-1-AP; proteintech)
Mouse anti-6xHis (HRP-66005; proteintech)
Mouse anti-GST (HRP-66001;proteintech)
Rabbit anti-beta-actin (20536-1-AP; proteintech)
Rabbit anti-PKM2 (4053S; Cell Signaling Technology)
Rabbit anti-p300 (54062; Cell Signaling Technology)
Rabbit anti-ATF4 (11815; Cell Signaling Technology)
Rabbit anti-CDKN1A/p21 (A11454;Abclonal)
Rabbit anti-PKM2 (A19102;Abclonal)
Mouse anti-SIRT6 (200499-6C9; ZENBO)
Rabbit anti-SOD1(A0274, Abclonal)
Rabbit anti-SOD2(A19576, Abclonal)
Rabbit anti-PPARγ(A11183, Abclonal)
Rabbit anti-PGC1α(A220995, Abclonal)
Rabbit anti-Caspase 3(A11040, Abclonal)

Custom made antibodies:
Rabbit anti-PKM2K433ac (custom made in Covance Inc.)
Rabbit anti-PKM2K305ac (Gift from Dr. Qunying Lei)

Validation

Commercially available antibodies:
Rabbit anti-histone H3 (ab1791; Abcam) has been validated for Western blots and ChIP in human and mouse (PMID30759223) (https://www.abcam.com/histone-h3-antibody-nuclear-loading-control-and-chip-grade-ab1791.html).
Rabbit anti-histone H3pT11 (ab5168; Abcam) has been validated for Western blots in human and mouse,ChIP in human (PMID29938647,PMID33500413) (https://www.abcam.com/histone-h3-phospho-t11-antibody-ab5168.html).
Mouse anti-FLAG M2 (F1804-1MG; Sigma-aldrich) has been validated for Western blots and ChIP of FLAG-tagged proteins in human (PMID30759223) (https://www.sigmaaldrich.com/catalog/product/sigma/f1804).
Mouse anti-Myc (60003-2-Ig; proteintech) has been validated for Western blots of recombinant protein and Myc-tagged proteins in human (https://www.ptgcn.com/products/MYC-Antibody-60003-2-Ig.htm).
Rabbit anti-PHGDH (14719-1-AP; Proteintech) has been validated for Western blots in human and mouse, IP in human (https://www.ptglab.com/Products/PHGDH-Antibody-14719-1-AP.htm).
Rabbit anti-GAPDH (10494-1-AP; proteintech) has been validated for Western blots in human (PMID30759223, PMID32663628) (https://www.ptglab.com/Products/GAPDH-Antibody-10494-1-AP.htm).
Rabbit anti-PSAT1 (10501-1-AP; proteintech) has been validated for Western blots in human (https://www.ptgcn.com/products/PSAT1-Antibody-10501-1-AP.htm).
Rabbit anti-PSPH (14513-1-AP; proteintech) has been validated for Western blots in human (https://www.ptglab.com/Products/PSPH-Antibody-14513-1-AP.htm).

Rabbit anti-PKM1 (15821-1-AP; proteintech) has been validated for Western blots in human (https://www.ptglab.com/Products/PKM1-specific-Antibody-15821-1-AP.htm).
Rabbit anti-p21 (10355-1-AP; proteintech) has been validated for Western blots in human (https://www.ptgcn.com/products/P21-Antibody-10355-1-AP.htm).
Rabbit anti-PCAF (13983-1-AP; proteintech) has been validated for Western blots in human (https://www.ptglab.com/Products/KAT2B-Antibody-13983-1-AP.htm).
Rabbit anti-SIRT1 (13161-1-AP; proteintech) has been validated for Western blots in human (PMID30930981)(http://www.ptgcn.com/products/SIRT1-Antibody-13161-1-AP.htm).
Mouse anti-6xHis (HRP-66005; proteintech) has been validated for Western blots of recombinant protein and 6xHis-tagged proteins (http://www.ptgcn.com/products/6-His,-His-Tag-Antibody-HRP-66005.htm)
Mouse anti-GST (HRP-66001;proteintech) has been validated for Western blots of recombinant protein and GST-tagged proteins (http://www.ptgcn.com/products/GST-Tag-Antibody-HRP-66001.htm)
Rabbit anti-beta-actin (20536-1-AP; proteintech) has been validated for Western blots in human and mouse (https://www.ptgcn.com/products/ACTB-Antibody-20536-1-AP.htm)
Rabbit anti-PKM2 (4053S; Cell Signaling Technology) has been validated for Western blots and IP in human (https://www.cellsignal.com/products/primary-antibodies/pkm2-d78a4-xp-rabbit-mab/4053).
Rabbit anti-p300 (54062; Cell Signaling Technology) has been validated for Western blots in human (https://www.cellsignal.com/products/primary-antibodies/p300-d2x6n-rabbit-mab/54062).
Rabbit anti-ATF4 (11815; Cell Signaling Technology) has been validated for Western blots in human, ChIP in mouse (https://www.cellsignal.cn/products/primary-antibodies/atf-4-d4b8-rabbit-mab/11815).
Rabbit anti-CDKN1A/p21 (A11454;Abclonal) has been validated for Western blots in human and mouse (https://abclonal.com.cn/catalog/A11454).
Rabbit anti-PKM2 (A19102;Abclonal) has been validated for Western blots in human and mouse (https://abclonal.com.cn/catalog/A19102).
Mouse anti-SIRT6 (200499-6C9; ZENBO) has been validated for Western blots in human (http://www.zen-bio.cn/prod_view.aspx?TypeId=127&Id=353991&FId=t3:127:3).
Rabbit anti-SOD1(A0274, Abclonal) has been validated for Western blots in mouse (https://abclonal.com.cn/catalog/A0274)
Rabbit anti-SOD2(A19576, Abclonal) has been validated for Western blots in mouse(https://abclonal.com.cn/catalog/A19576)
Rabbit anti-PPARγ(A11183, Abclonal) has been validated for Western blots in mouse(https://abclonal.com.cn/catalog/A11183)
Rabbit anti-PGC1α(A220995, Abclonal) has been validated for Western blots in mouse(https://abclonal.com.cn/catalog/A20995)
Rabbit anti-Caspase-3(A11040, Abclonal) has been validated for Western blots in mouse(https://abclonal.com.cn/catalog/A11040)

Custom made antibodies:
Rabbit anti-PKM2K433ac (custom made in Covance Inc.) has been validated for Dot blots in Extended Data Fig. 3k.
Rabbit anti-PKM2K305ac has been validated in PMID: 21700219.

Eukaryotic cell lines

Policy information about cell lines and Sex and Gender in Research

Cell line source(s): HeLa and HEK293T cells were obtained from the American Type Culture Collection (ATCC). HUVEC cells were purchased from ScienCell Research Laboratories (Catalog #8000). All cell lines were cultured for no more than 2 months and their morphology was confirmed periodically to avoid cross-contamination.

Authentication: HeLa and HEK293T cells were authenticated through short tandem repeat (STR) analysis. HUVEC cells were authenticated by ScienCell Research Laboratories.

Mycoplasma contamination: All cell lines were regularly tested for mycoplasma contamination. The results showed that all cell lines have no mycoplasma contamination.

Commonly misidentified lines (See ICLAC register): No commonly misidentified cell lines were used.

Animals and other research organisms

Policy information about studies involving animals; ARRIVE guidelines recommended for reporting animal research, and Sex and Gender in Research

Laboratory animals: Our research complies with all relevant ethical regulations. All procedures were approved by the Animal Care and Use Committee of Wuhan Sports University and Hubei University. Mice in a C57BL/6 background (male, female) were used in this study.

For natural ageing experiments, 3-month-old mice (female) in a C57BL/6 background were purchased from Beijing Vital River Laboratory Animal Technology Co., Ltd. (Beijing, China). The mice were randomly divided into five groups (six mice per group) and housed under a 12-h light and 12h dark cycle with an ambient temperature of 22 ± 2 °C and humidity of 55 ± 10% for up to 26 months. Water and standard chow were provided ad libitum following the regulations and guidelines of Wuhan Sports University and Hubei University. Mice in each group were killed, the heart, liver, kidney and spleen tissues were collected at 3, 6, 12, 20 and 26 months.

For adenovirus infection experiments, 18-month-old mice (male, female) in a C57BL/6 background were purchased from Beijing Vital River Laboratory Animal Technology Co., Ltd. (Beijing, China). The mice were housed under a 12-h light and 12-h dark cycle. Water and standard chow were provided ad libitum following the regulations and guidelines of Wuhan Sports University. The mice were divided into three groups (23-24 mice per groups, half male and female). After one week of acclimatization, mice were then injected with 100 μl 5×10^11 viral genomes of AAV1-ICAM2-Control (AAV-Ctrl), AAV1-ICAM2-PHGDH (AAV-PHGDH) or AAV1-ICAM2-PKM2 (AAV-PKM2) particles through tail vein injection. AAV1-ICAM2-Control (AAV-Ctrl), AAV1-ICAM2-PHGDH (AAV-PHGDH) or AAV1-

	ICAM2-PKM2 (AAV-PKM2) particles were prepared and purchased from Taitool Bioscience (Shanghai, China). The mice used in this study were examined by the investigator in a double-blinded manner. One week later, the mice were injected with the same dose. The body weight of mice was measured every three days. After four weeks for injection, the ageing-related phenotypes were measured. All procedures were approved by the Animal Care and Use Committee of Wuhan Sports University and Hubei University. The body weight of mice was measured every three days.
Wild animals	The study did not involve wild animals.
Reporting on sex	We specified both males and females used in this study. There was no marked difference for male and female mice on heart weight, heart rate for male and female. For LV ejection fraction, LV fractional shortening and exercise capability, over-expression of PHGDH and PKM2 displayed a little better beneficial effect in female mice than male mice.
Field-collected samples	The study did not involve samples collected from the field.
Ethics oversight	Animal procedures complied with all relevant ethical regulations and were approved by the Animal Care and Use Committee of Wuhan Sports University and Hubei University.

Note that full information on the approval of the study protocol must also be provided in the manuscript.
